# Case Report: Acute kidney failure leading to permanent haemodialysis due to hyperoxaluria following one-anastomosis gastric bypass-related rapid weight loss.

**DOI:** 10.12688/f1000research.22109.2

**Published:** 2020-05-29

**Authors:** Angelo Miranda, Andrea Rosato, Andrea Costanzi, Lucia Pisano, Sara Colzani, Sara Auricchio, Giulio Mari, Pietro Achilli, Dario Maggioni

**Affiliations:** 1 General Surgery Departement, Desio Hospital, Desio, Italy, 20843, Italy; 2 Nephrological Unit, Desio Hospital, Desio, Italy, 20843, Italy; 3 Univerity of Milan, Residency in general Surgery, Milano, Italy, 20100, Italy

**Keywords:** OAGB, kidney failure, Calcium oxalate, weight loss

## Abstract

The one-anastomosis gastric bypass (OAGB) has been proven to provide good weight loss, comorbidity improvement, and quality of life with follow-up longer than five years. Although capable of improving many obesity-related diseases, OAGB is associated with post-operative medical complications mainly related to the induced malabsorption. A 52-year-old man affected by nephrotic syndrome due to a focal segmental glomerulosclerosis underwent OAGB uneventfully. At three months post-surgery, the patient had lost 40kg, reaching a BMI of 32. The patient was admitted to the nephrology unit for acute kidney injury with only mild improvement in renal function (SCr 9 mg/dl); proteinuria was still elevated (4g/24h), with microhaematuria. A renal biopsy was performed: oxalate deposits were demonstrated inside tubules, associated with acute and chronic tubular and interstitial damage and glomerulosclerosis (21/33 glomeruli). Urinary oxalate levels were found to be elevated (72mg/24h, range 13-40), providing the diagnosis of acute kidney injury due to hyperoxaluria, potentially associated to OAGB. No recovery in renal function was observed and the patient remained dialysis dependent. Early and rapid excessive weight loss in patients affected by chronic kidney insufficiency could be associated with the worsening of renal function. Increased calcium oxalate levels associated with OAGB-related malabsorption could be a key factor in kidney injury.

## Introduction

Bariatric surgery is known to be the most effective means of inducing durable weight loss in obese populations ^1– 3^. In particular, bariatric malabsorptive procedures are effective metabolic therapies capable of improving many obesity-related diseases ^4, 5^.

Among bariatric procedures claiming to be effective in providing a metabolic improvement in obese patients, the one-anastomosis gastric bypass (OAGB) has been proven to provide good weight loss, comorbidity improvement, and quality of life even with follow-up longer than five years ^6^. Despite the positive reported results in terms of surgical outcomes and effectiveness in reducing several obesity-related diseases, OAGB is associated with post-operative medical complications, mainly related to the induced malabsorption ^7^. Focusing on renal function, bariatric surgery is capable of being a protective factor against renal failure ^8, 9^; however, the association between induced malabsorption and particular renal syndromes can significantly alter the metabolism of numerous serum metabolites, leading to direct kidney damage. Renal syndroms involving the glomerulus asset may express themselves towards the different metabolites with variable intensity. ^10^. When reported, such alterations of the metabolic framework tend to appear a relatively long time after surgery and are described as a late complication of weight loss ^11^. However, if these alterations settle on an impaired renal calcium oxalate metabolism, they could aggravate and accelerate the kidney injury.

Here, we report a rare case of acute renal failure occurring three months after laparoscopic OAGB caused by hyperoxaluria associated with rapid and early excessive weight loss.

## Case presentation

A white Italian 52-year-old man employed at an insurance company underwent OAGB in February 2019 for stage III morbid obesity (135kg, BMI 45).

Past medical history was characterized by pathological obesity, type 2 diabetes, obstructive sleep apnoea syndrome and nephrotic syndrome due to focal segmental glomerulosclerosis. In 2011, at the time of the initial kidney biopsy, his weight was 110kg, his blood pressure and kidney function were normal, and proteinuria was 9g/24h. He was then treated with steroids and after five months cyclosporin was added, leading to complete remission of the nephrotic syndrome. He was also treated with renin-angiotensin-system blockers. In 2015, his weight was 125kg and his serum creatinine (SCr) and proteinuria began to rise, until 2018 when his weight was 134kg, SCr was 1.6mg/dl and proteinuria 4.8g/24h, without nephrotic syndrome. The patient had tried to follow a hypocaloric and hypoproteic diet with no success. The worsening of proteinuria was interpreted as secondary to obesity; in the meantime, steroids and cyclosporin were both contraindicated.

The patient was then referred to a surgeon for bariatric surgery. Laparoscopic OAGB was pleanned. In February 2019, the surgical procedure was carried out uneventfully under general anaesthesia.. The gastric pouch was fashioned with an EndoGIA linear stapler and the length of the bilio-pancreatic loop was 200 cm from Treitz ligament after measuring the entire small bowel. The gastric sleeve was fashioned with a 60 mm stapler (six charges) and Seamguard. The operating surgeon was highly experienced in laparoscopic bariatric surgery, having performed more than five hundred procedures. The patient was discharged on day three after surgery after the methylene blue test did not reveal any signs of staple line leak. Follow-up was done monthly for the first six months after surgery with outpatient visits. The patient’s SCr was unvaried. In March 2019, the patient’s body weight was 114.5 kg; his sCr was 2.12 mg/dl in March and 2.23 mg/dl in April 2019. In May 2019, his body weight was 95 Kg. His water and food intake were very poor; he had stopped taking vitamin and citrate supplements and his SCr had raised up to 16 mg/dl. The excess weight loss was 64% at two months; at three months after surgery, the patient had lost 40kg, reaching a BMI of 32.

The patient was admitted to our nephrology unit for acute kidney injury. His blood pressure was 140/80 mmHg, heart rate was 77 bpm and urine output was about 2000 ml/day. On admission, Scr was 16.6 mg/dl, urea was 235 mg/dl, sodium and potassium levels were normal despite metabolic acidosis (HCO _3_ 16 mmol/l), proteinuria was 3 g/day and mild microhaematuria was detected using a urine dipstick. Renal ultrasound was normal. The patient was given fluids (2 litres of polysaline solution intravenously for 10 days and an oral intake of water of 1.5 litres) with only mild improvement in renal function (SCr 8mg/dl). A new renal biopsy was performed; the puncture was ultrasound-guided using 16g × 20 mm Bard Monopty needles and two specimens were obtained for histologic examination. Oxalate deposits were demonstrated inside tubules, associated with acute and chronic tubular and interstitial damage and glomerulosclerosis (21/33 glomeruli). Urinary oxalate level was then found to be elevated (72mg/24h, range 13–40), providing the diagnosis of acute kidney injury due to hyperoxaluria potentially associated to OAGB.

In July 2019, renal replacement therapy was started. Online hemodiafiltration was administered through a central venous catheter and the treatment was performed three times a week for four hours without ultrafiltration. The patient was also treated with oral sodium bicarbonate (NaHCO _3_, 2 g/day), potassium and magnesium citrate (2.56 g/day), calcium carbonate (2.5g/day) and sevelamer (2.4 g/day). Renal function was assessed monthly by creatinine and urea clearance tests, but no recovery in renal function was observed and the patient remained dialysis dependent (creatinine clearance 11 ml/min, urea clearance 4 ml/min in January 2020). The patient’s body weight is now steady at 84Kg.

## Discussion

Kidney failure can complicate the post-operative course after bariatric surgery. Its aetiology can be multifactorial and complex in obese populations. Pre-existing factors such as diabetes, hypertension, and chronic kidney disease (CKD) can play a role in worsening the renal insult during the early post-operative period ^12, 13^. In addition to dehydration, which is the main cause of acute kidney failure after bariatric surgery, the alteration of several metabolic assets induced by a rapid and consistent weight loss can become a key factor in triggering kidney failure ^14^. One metabolite potentially involved in the acute onset of kidney failure but rarely associated to it, is calcium oxalate ^15^.

The reasons for the onset of hyperoxaluria are not yet completely understood. There are various hypotheses: one of the main reasons seems to be the malabsorption of lipids since, in this condition, the calcium found inside the intestinal lumen tends to bind to fatty acids instead of forming insoluble precipitates of calcium oxalate; the oxalate thus remains in a soluble state and can be reabsorbed by the ileal walls ^16^. Another possible explanation is the increase in bile secretion into the colon due to the lack of intestinal reabsorption of bile salts, with consequent increase in the permeability of the colic walls due to a decrease in the function of the epithelial barrier. Finally, another hypothesis is the decrease in colonization of the colon by oxalate-metabolizing bacteria such as *Oxalobacter formigenes*, able to metabolize oxalate ^17^.

The association between excessively rapid weight loss and renal damage due to calcium oxalate is not widely documented in the literature and therefore remains a hypothesis based on a valid rational ^18^.

Bariatric surgery is able to reduce the most dangerous risk factors for end stage renal disease such as hypertension and diabetes, but post-operative complications in CKD patients are slightly higher than the general population and surgeons must be aware of oxalate nephropathy, because it’s a rare but often irreversible cause of acute kidney injury ^19, 20^.

Patient with pre-existing chronic kidney disease may be considered to be higher risk for secondary oxalate nephropathy but this has not yet been established. Monitoring the 24-hour urinary oxalate excretion rate might be a useful tool to prevent oxalate nephropathy in high risk patients. For such patients, a careful selection of the right bariatric procedure (malabsorptive vs restrictive) should therefore be performed ^21^.

The clinical case that we report here underlines a clinical-pathological hypothesis about the correlation between OAGB, rapid weight loss and calcium oxalate-related renal damage. Deepening into the pathological pattern involved is certainly not simple. As proof of this we have to acknowledge that also some dietary treatments lead to a rapid weight loss, yet an association with the increase in calcium oxalate levels is not reported. In view of this the routine dosage of calcium oxalate in patients with nephrotic syndrome due to focal segmental glomerulosclerosis undergoing OAGB before and after surgery could better clarify the possible correlation involved.

## Conclusions

The early and rapid excessive weight loss in patients affected by chronic kidney insufficiency could be associated with the worsening of renal function. Increased calcium oxalate levels associated with OAGB-related malabsorption could be a key factor in kidney injury.

## Consent

Written informed consent for the publication of their clinical details and clinical images and video was obtained from the patient.

## Data availability

### Underlying data

All data underlying the results are available as part of the article and no additional source data are required.

### Extended data

Zenodo: Acute kindey failure leading to permanent hemodalysis due to hyperoxaluria following OAGB related rapid weight loss. Case report. https://doi.org/10.5281/zenodo.3609350 ^22^

This project contains the following extended data:

- Surgical procedure video (in MP4 file format)
- Creatinine levels pre-surgery (in JPG format)
- Creatinine levels post-surgery (n JPG format)

Data are available under the terms of the Creative Commons Attribution 4.0 International license (CC-BY 4.0).
