# Supplementary material for: Case Report: Acute kidney failure leading to permanent haemodialysis due to hyperoxaluria following one-anastomosis gastric bypass-related rapid weight loss
Source: F1000Res. 2020 May 29;9:155. Originally published 2020 Feb 26. [Version 2] doi: 10.12688/f1000research.22109.2 (PMC7265574; doi:10.12688/f1000research.22109.2)
Supplement: Peer review history

# Peer review: Reviewer response for version 2

**DOI:** 10.5256/f1000research.26931.r64072

**Published:** 2020-06-01

**Authors:** Mohammad Kermansaravi

**Affiliations:** 1 Minimally Invasive Surgery Research Center, Iran University of Medical Sciences, Tehran, Iran

The manuscript is now accepted.

Are enough details provided of any physical examination and diagnostic tests, treatment given and outcomes?

Partly

Is the case presented with sufficient detail to be useful for other practitioners?

Partly

Is sufficient discussion included of the importance of the findings and their relevance to future understanding of disease processes, diagnosis or treatment?

Partly

Is the background of the case’s history and progression described in sufficient detail?

Partly

Reviewer Expertise:

Bariatric surgery, metabolic surgery, dvanced laparoscopic surgery

I confirm that I have read this submission and believe that I have an appropriate level of expertise to confirm that it is of an acceptable scientific standard.

# Peer review: Reviewer response for version 1

**DOI:** 10.5256/f1000research.24383.r63316

**Published:** 2020-05-18

**Authors:** Mohammad Kermansaravi

**Affiliations:** 1 Minimally Invasive Surgery Research Center, Iran University of Medical Sciences, Tehran, Iran

It's a well written manuscript, but I have some suggestions:

1. In abstract and introduction, it's better write " one-anastomosis gastric bypass (OAGB)" instead of " one-anastomosis laparoscopic gastric bypass (OAGB)"
2. In case presentation, you mentioned that the patient underwent sleeve gastrectomy! Sleeve or OAGB? Please clarify the surgical technique.
3. There is not any strong evidence about rapid weight loss related nephropathy. How do you result in? Please clarify.
4. How do you justify the relation between nephropathy and OAGB? Rapid weight loss if imaginary has a relation with rapid weight loss, it could happen in any type of weight loss therapies.

Are enough details provided of any physical examination and diagnostic tests, treatment given and outcomes?

Partly

Is the case presented with sufficient detail to be useful for other practitioners?

Partly

Is sufficient discussion included of the importance of the findings and their relevance to future understanding of disease processes, diagnosis or treatment?

Partly

Is the background of the case’s history and progression described in sufficient detail?

Partly

Reviewer Expertise:

Bariatric surgery, metabolic surgery, dvanced laparoscopic surgery

I confirm that I have read this submission and believe that I have an appropriate level of expertise to confirm that it is of an acceptable scientific standard, however I have significant reservations, as outlined above.

# Response

**Published:** 2020-05-26

**Authors:** Giulio Mari

**Affiliations:** Desio Hospital, Italy

We corrected the OAGB definition as indicated.

We better described the OAGB procedure in the case presentation.

As requested by the reviewer we clarified the possible correlation between OAGB, rapid weight loss and kidney failure due to calcium oxalate.

The case report that we report describes a kidney failure following OAGB and rapid weight loss. The only change in the renal function across surgery was the calcium oxalate levels increase. We therefore make an hypothesis based on a pathophysiological rationale that will need further analysis to be proven.

# Peer review: Reviewer response for version 1

**DOI:** 10.5256/f1000research.24383.r60620

**Published:** 2020-02-28

**Authors:** Matteo Uccelli

**Affiliations:** 1 Department of Surgery, S.I.C.OB. (Italian Society of Bariatric Surgery) Referral Center for Bariatric Surgery, San Donato Group, Policlinico San Marco, Zingonia-Osio Sotto, Italy

- The case report described here presents a useful suggestion in deciding which bariatric procedure should be chosen in nephropathic patients.
- The patient description is well explained and the discussion is well written as it establishes a sort of guide line that deserves to be taken into consideration.
- Kidney function in obese patients is a main issue and a main concern. Analyzing which kind of pathological disorder lays underneath kidney insufficiency should be done before choosing the type of bariatric procedure.

Are enough details provided of any physical examination and diagnostic tests, treatment given and outcomes?

Yes

Is the case presented with sufficient detail to be useful for other practitioners?

Yes

Is sufficient discussion included of the importance of the findings and their relevance to future understanding of disease processes, diagnosis or treatment?

Yes

Is the background of the case’s history and progression described in sufficient detail?

Yes

Reviewer Expertise:

bariatric surgery

I confirm that I have read this submission and believe that I have an appropriate level of expertise to confirm that it is of an acceptable scientific standard.

## References

[1] : Analyzing the Impact of Bariatric Surgery in Kidney Function: a 2-Year Observational Study. *Obes Surg*.29(1) : 10.1007/s11695-018-3508-1 197-206 10.1007/s11695-018-3508-1 30238213
